# Supplementary material for: Work Ability and Job Survival: Four-Year Follow-Up
Source: Int J Environ Res Public Health. 2019 Aug 28;16(17):3143. doi: 10.3390/ijerph16173143 (PMC6747402; doi:10.3390/ijerph16173143)
Supplement: Supplementary file 1 [file ijerph-16-03143-s001.pdf]

SUPPLEMENTARY INFORMATION 01

"Work ability and job survival: four-year follow-up"

Maria Carmen Martinez, Frida Marina Fischer

Work ability index

The Work Ability Index (WAI) is an instrument that allows assessing work ability based on the workers' self-perception. Work ability is defined as the workers' capacity to perform work tasks as a function of the job demands and the workers' state of health and physical and mental ability [Tuomi et al., 2006].

WAI comprises 10 questions corresponding to seven dimensions:

Dimension	Questions	Points
1. Current work ability compared with the lifetime best	1. Assume that your work ability at its best has a value of 10 points. How many points would you give your current work ability?	0 – 10
2. Work ability in relation to the demands of the job	2. How do you rate your current work ability with respect to the physical demands of your work?	1 – 5
	3. How do you rate your current work ability with respect to the mental demands of your work?	1 – 5
3. Number of current diseases diagnosed by a physician	4. In the following list, mark your current diseases or injuries. Also indicate whether a physician has diagnosed or treated these diseases (list of 51 diseases)	1 – 7
4. Estimated work impairment due to diseases	5. Is your illness or injury a hindrance to your current job?	1 – 6
5. Sick leave during the past year (12 months)	6. During the last 12 months: how many whole days have you been off work because of:	1 - 5
6. Own prognosis of work ability two years from now	7. Do you believe, according to your present state of health, that you will be able to do your current job two years from now?	1 - 7
7. Mental resources	8. Considering the last three months: Have you been able to enjoy your regular daily activities?	0 - 4
	9. Considering the last three months: Have you been active and alert?	0 - 4
	10. Considering the last three months: Have you felt yourself to be full of hope about the future?	0 - 4

The results of the seven dimensions provide a measure of work ability that ranges from 7 to 49. The higher the value, the better the work ability [Tuomi et al., 2006]. WAI was validated for use in Brazil [Martinez et al., 2009].

The initial and final scores on WAI was classified as excellent, good, moderate or poor work ability according to the criteria formulated by Kujala et al. [2005] and Tuomi et al. [2005]:

- Age $\geq$ 35 years old - poor: 7.0 to 27.9; moderate: 28.0 to 16.9; good: 37.0 to 43.9; excellent: 44.0 to 49.0 [Tuomi et al., 2005];
- Age < 35 years old - poor: 7.0 to 36.9; moderate: 37.0 to 40.9; good: 41.0 to 44.9; excellent: 45.0 to 49.0 [Kujala et al., 2005];

References:

Kujala V, Remes J, Ek E, Tammelin T, Laitinen J. 2005. Classification of Work Ability Index among young employees. Occup Med 55(5):399–401.

Martinez MC, Latorre MRDO, Fischer FM. 2009. Validity and reliability of the Brazilian version of the Work Ability Index questionnaire. Rev Saúde Pública 43(3):55-61

Tuomi K, Ilmarinen J, Jahkola A, Katajarinne L, Tulkki A. 2005. Índice de capacidade para o trabalho, São Carlos: EduFSCar.

Tuomi K, Ilmarinen J, Jahkola A, Katajarinne L, Tulkki A. 2006 Work Ability Index, Helsinki: Finnish Institute of Occupational Health.

SUPPLEMENTARY INFORMATION 02

Work ability and job survival: four-year follow-up

Maria Carmen Martinez, Frida Marina Fischer

Work ability index results from the studied population

Study population distribution (nº and%) according to the WAI - Work Ability Index dimensions score, Workers Hospital, São Paulo, 2008.

Dimension	Points											
	0	1	2	3	4	5	6	7	8	9	10	Total
Current work ability compared to the lifetime best	--<br>(--)	2<br>(0,2)	1<br>(0,1)	1<br>(0,1)	--<br>(--)	21<br>(2,0)	16<br>(1,5)	84<br>(8,1)	295<br>(28,4)	330<br>(31,8)	287<br>(27,7)	1.037<br>(100,0)
Work ability in relation to the job demand	--<br>(--)	--<br>(--)	--<br>(--)	--<br>(--)	3<br>(0,3)	4<br>(0,4)	49<br>(4,7)	110<br>(10,6)	401<br>(38,7)	226<br>(21,8)	244<br>(23,5)	1.037<br>(100,0)
Current diseases self-reported and diagnosed by a physician	*	73<br>(7,0)	51<br>(4,9)	80<br>(7,7)	156<br>(15,0)	257<br>(24,8)	*	420<br>(40,5)	*	*	*	1.037<br>(100,0)
Estimated work impairment due to diseases	*	1<br>(0,1)	4<br>(0,4)	11<br>(1,1)	132<br>(12,7)	197<br>(19,0)	692<br>(66,7)	*	*	*	*	1.037<br>(100,0)
Sick leave	*	12<br>(1,2)	21<br>(2,0)	84<br>(8,1)	325<br>(31,3)	595<br>(57,4)	*	*	*	*	*	1.037<br>(100,0)
Own prognosis of work ability	*	40<br>(3,9)	*	*	94<br>(9,1)	*	*	903<br>(87,1)	*	*	*	1.037<br>(100,0)
Mental resources	*	1<br>(0,1)	50<br>(4,8)	295<br>(28,4)	691<br>(66,6)	*	*	*	*	*	*	1.037<br>(100,0)

* Nonexistent points at dimension score
